# Using multiple short epochs optimises the stability of infant EEG connectivity parameters

**DOI:** 10.1038/s41598-020-68981-5

**Published:** 2020-07-29

**Authors:** Rianne Haartsen, Bauke van der Velde, Emily J. H. Jones, Mark H. Johnson, Chantal Kemner

**Affiliations:** 1 Department of Psychological Sciences (BMA), Centre for Brain and Cognitive Development, Birkbeck College, University of London, Malet Street, London, WC1E 7HX UK; 2 Department of Experimental Psychology, Helmholtz Institute, Utrecht University, Heidelberglaan 1, 3584 CS Utrecht, The Netherlands; 3 Department of Developmental Psychology, Utrecht University, Utrecht, The Netherlands; 4 Department of Psychiatry, Brain Center Rudolf Magnus, University Medical Center Utrecht, Heidelberglaan 100, 3508 GA Utrecht, The Netherlands; 5 Department of Psychology, University of Cambridge, Cambridge, UK

**Keywords:** Cognitive neuroscience, Paediatric research

## Abstract

Atypicalities in connectivity between brain regions have been implicated in a range of neurocognitive disorders. We require metrics to assess stable individual differences in connectivity in the developing brain, while facing the challenge of limited data quality and quantity. Here, we examine how varying core processing parameters can optimise the test–retest reliability of EEG connectivity measures in infants. EEG was recorded twice with a 1-week interval between sessions in 10-month-olds. EEG alpha connectivity was measured across different epoch lengths and numbers, with the phase lag index (PLI) and debiased weighted PLI (dbWPLI), for both whole-head connectivity and graph theory metrics. We calculated intra-class correlations between sessions for infants with sufficient data for both sessions (N’s = 19–41, depending on the segmentation method). Reliability for the whole brain dbWPLI was higher across many short epochs, whereas reliability for the whole brain PLI was higher across fewer long epochs. However, the PLI is confounded by the number of available segments. Reliability was higher for whole brain connectivity than graph theory metrics. Thus, segmenting available data into a high number of short epochs and calculating the dbWPLI is most appropriate for characterising connectivity in populations with limited availability of EEG data.

## Introduction

Neurological and psychiatric disorders have been associated with disruptions or atypicalities in brain networks^1^. Early environmental and genetic influences may have cascading effects that converge to affect trajectories of brain development^2^. Given the substantial changes in white matter, brain structure and connectivity during the first few years of life^3,4^, studying functional whole brain connectivity can provide insight into the integrity of early brain development. Examining how individual variability in infant brain connectivity relates to later outcomes can reveal the atypicalities in early brain development that presage later diagnoses of neurodevelopmental disorders^1,5,6^, and the early effects of interactions between genetic and environmental risk factors^7^. Furthermore, this work has potentially important implications for disorder identification within a global mental health framework^8,9^.

Alterations in brain connectivity have been associated with variation in candidate gene studies and genome wide association studies^7^. Environmental risk factors have also been linked to altered brain connectivity, spanning factors present at prenatal periods (i.e. maternal mood disorders, substance abuse, psychosocial factors^7,10,11^), perinatal periods (i.e. prematurity and early brain injury^7,12–15^), and during childhood (i.e. adverse events and socioeconomic status^7,16^). During infancy, brain connectivity shows age related increases where networks become more efficient and long-range connections become stronger with age^17,18^. Atypical brain connectivity patterns during early development have been associated with developmental disorders such as autism spectrum disorder, attention deficit/hyperactivity disorder, and schizophrenia^1,19–24^. Finally, individual variability in brain connectivity has been associated with variability in cognitive skills. For example, increased thalamocortical connectivity at term age in preterm neonates has been linked to higher general cognitive developmental levels at age 2 years^25^. Increased thalamocortical connectivity at 1 year of age associated with better working memory abilities and higher levels of general cognitive development at 2 years of age^26^. In 14-month-old infants who received a diagnosis of autism spectrum disorder, elevated EEG alpha connectivity predicted higher severity of restricted and repetitive behaviours at 3 years of age^21,27^. Lastly, reduced connectivity strengths in the cortico-basal ganglia-thalamo-cortical loop was associated with poorer concurrent socio-cognitive performance in 6-year-olds who were born extremely premature or after intrauterine growth restriction^28^.

If individual differences in brain connectivity are mechanistically linked to stable developmental traits, one would expect that these individual differences in brain connectivity should also show a degree of intra-individual stability. For example, restricted and repetitive behaviours in toddlerhood are stable across 13 months in 2–5-year-olds^21,27,29^. Given that neural connectivity at 12 months predicts repetitive behaviours at age 2^21,27^, individual differences in infant brain connectivity should exhibit a degree of stability within individuals. At least some degree of persistence over time would likely be necessary for either the individual differences in connectivity to underpin differences in behaviour at the later timepoint, or for individual differences measured in infants with a relatively heterogenous age span to have sufficient predictive value for later behaviour. This is particularly relevant for developmental studies in neurodevelopmental disorders who aim to identify early factors of atypical development and examine the stability of these factors across different time windows during infancy and toddlerhood (e.g.^30^).

Whole brain connectivity can be measured using EEG (electroencephalography). This method allows high temporal resolution, which allows for the investigation of how brain regions communicate^31,32^. The method is scalable to different contexts and settings, and suitable for different developmental populations due to its relatively low movement restrictions^33^. These advantages make EEG an excellent method to measure emerging networks and their characteristics. However, there are some outstanding questions that still need to be addressed. In order to be feasible as a robust measure for predicting later outcomes, infant EEG connectivity metrics should have low measurement error. Further, individual differences should persist at least briefly in development (such that the same set of measures can be taken in a group of infants of a similar age), rather than fluctuating on a day to day basis. Both these features are encapsulated in the concept of ‘test–retest reliability’: the degree to which scores in a test are consistent between two administrations. Previous work with EEG indicates that infant brain activity can be reliably measured: for example, a previous EEG study demonstrated good reliability of amplitudes of event related potentials in 10-month-old infants tested with an interval of 1 week^34^. Here, we ask: can we reliably measure brain networks in infants at a similar interval? What network characteristics can we measure reliably? How can we measure these characteristics in an optimal way?

Adult test–retest studies show reliability of EEG connectivity estimates varies with calculation methods, epoch numbers and durations, network characteristics, and frequency bands, among other factors^35–45^. One example of an EEG connectivity calculation method is the phase lag index (PLI), which reflects the consistency of the lag in phase between 2 signals^44,46^. The debiased weighted phase lag index (dbWPLI) calculates the consistency of the phase lag between signals also, but assigns smaller weights to smaller phase lags that are likely influenced by noise^44^. Both methods come with their own strengths and weaknesses: the PLI is affected by epoch number, and overestimates connectivity when calculated across a small number of epochs. In contrast, the dbWPLI corrects for this inflation, and is more robust to noise. However, the robustness to noise from small phase lags also leads to an underestimation of short-range connectivity from the dbWPLI, which is not present for the PLI-based EEG connectivity estimates. In addition, local network characteristics such as the normalised clustering coefficient are more reliable than global network characteristics such as the normalised path length and small-worldness index^35,38^. It remains relatively unknown whether similar patterns hold for infants.

It is possible that a different pattern holds for infants compared to adults with regards to test–retest reliability for different methods^47^. First, infants may exhibit a less stable pattern of network connectivity as networks are still emerging^18^. Different epoch numbers and lengths may be needed to reliably assess infant networks compared to adult brain networks. Second, the collection of sufficient artefact-free data is a major challenge in young infants. The quantity of artefact-free data segments differs significantly between different populations over the life span. Adults are more compliant and better able to follow verbal instructions than infants or young children. Infants are more likely to move around and have shorter attention spans than adults resulting in fewer and shorter segments of clean data. While in a perfect world the inclusion of long segments would provide more reliable results, in reality the amount of EEG data available per infant is finite. This means there is a trade-off between numbers and durations of epochs: an EEG data segment can be cut into a high number of short epochs, or a low number of long epochs. The pragmatic question that arises here is which parameters of epoch length and numbers would provide the most reliable EEG connectivity estimates in infant research given the finite amounts of available data it is possible to collect.

In our recent study, we evaluated the reliability of network characteristics across different frequency bands in 60 typically developing infants^48^. EEG was recorded while 10-month-olds watched dynamic naturalistic stimuli as part of a larger battery. Reliability of ERPs in the same infants has previously been reported in^34^. The session was repeated after a 1-week delay. Network characteristics were based on PLI calculations across 20 5-s epochs. Whole brain connectivity displayed higher reliability values than the normalised clustering coefficient, which in turn exhibited higher reliability values than the normalised path length. In addition, reliability values differed across frequency bands: highest values were found for measures between 3 and 9 Hz (theta and alpha band). This is consistent with adult studies showing that theta and alpha band frequencies are most reliable during resting state paradigms^38,39,41,45,49^.

The conclusions from our previous reliability study were based on data segmented and analysed in a specific way: PLI-based connectivity estimates from 20 5-s epochs. The aim of the current study is to examine how *different* numbers and durations of epochs affect test–retest reliability of the dbWPLI- and PLI-based connectivity metrics in young infants. To this end, we analysed the data from our previous study while varying the quantity and lengths of data segments and deriving the phase lag indices from Fourier coefficients^44^. We then calculated intra-class correlations between the connectivity measures of session 1 and 2 for each combination of number and duration of epochs and explored the pattern ICCs for varying epoch numbers and lengths. This allows us to address the practical question of how data should be prepared for connectivity analysis.

## Material and methods

### Participants

This study was part of a larger investigation that focussed on the test–retest reliability of behavioural, eye tracking, and EEG measures across 2 sessions separated by a 1 week delay (mean 7.8, range 2–20 days for the included infants). A delay of 1 week was selected to minimise the effects of repetition on infant attention and responses^50^ and to encompass a degree of developmental stability. Shorter intervals may lead to data loss (see section Attrition rates in Supplementary Information). Longer intervals may encompass significant developmental change, confounding interpretation. The study was conducted at the Kinder Kennis Centrum at Utrecht University, The Netherlands, where a team of trained and experienced researchers and research assistants collected the data. The medical ethical committee of the University Medical Center Utrecht approved the study (application number: 14-221), and all methods were carried out in accordance with the relevant guidelines and regulations.

Families with infants aged around 10 months were invited to participate in the study in writing (home addresses were shared with the research centre by the communal register of the cities within the Utrecht province). Upon arrival at the lab, legal guardians of the infants (parents/caregivers) received information about the procedure of the study and gave signed informed consent. After the session had finished, they received 30 euros and a toy for the participating infant as an incentive. The session was repeated after 1 week. EEG data for the first session were available for 73 infants, and for the second session for 64 infants (the remaining 9 families did not want to return for a second session). EEG data and participants are identical to those reported in the study by Van der Velde et al.^48^.

**Figure 1:**
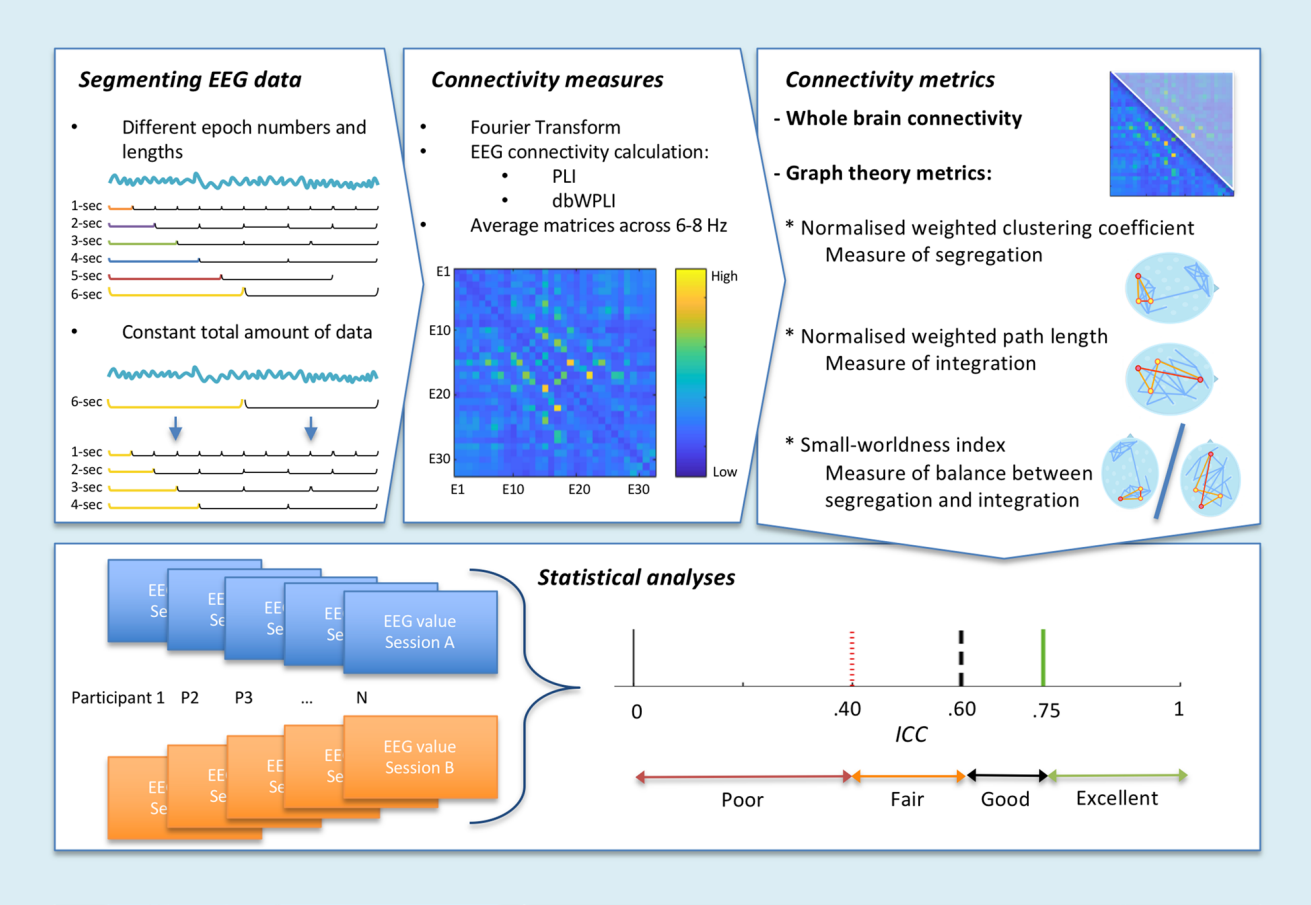
Overview of the methods. Clean EEG data were segmented in different epoch lengths. After randomly selecting different numbers of epochs, connectivity matrices were calculated with the PLI and dbWPLI methods, and averaged across 6–8 Hz. Finally, connectivity metrics were derived from the matrices. Reliability was calculated with the intra-class correlation (ICC) for the extracted connectivity metrics from different methods from both sessions.

After data cleaning, different subsamples of the data were used for the analyses in order to include the maximal number of participants with specific amounts of data available. First, we selected the alpha frequency band based on visual inspection of data from the first session in the 73 infants (35 males, *M*_Age_ = 302 days, *sd*_Age_ = 13, range 272–344 days). Second, we included 3 different subsamples for analyses including long epochs, short epochs, and with constant amounts of data (see “Selection of epoch lengths and numbers” and Fig. 1 for an overview of the methods).

### Experimental procedure

The EEG task consisted of the presentation of naturalistic dynamic videos: 5 vignettes of women singing Dutch nursery rhymes (recorded in The Netherlands after^51^), and 6 vignettes of moving toys^51^ (60 s duration each). Videos were presented 3 times as part of a larger EEG battery, resulting in a total duration of 6 min. Infants were seated in a high chair in front of the stimulus screen, with their parents sitting behind them. A curtain separated the participants and stimulus screen from the experimenter and recording screen to avoid the infants being distracted by the experimenter.

The EEG signal was recorded with a 32 electrode Biosemi ActiveTwo system at a sampling rate of 2048 Hz (a layout can be found in the Supplementary Information online). The Common Mode Sense (CMS) and Driven Right Leg (DRL) were used as active ground signal. Two external electrodes on the left and right mastoid and one electrode under the eye were recorded as well. The EEG session was recorded with a video camera.

### EEG data cleaning and segmenting

Raw EEG data were preprocessed using Matlab (versions 2015a and 2017a, Natick, MA, USA), and Fieldtrip (a toolbox for MEG/EEG data processing, available at https://www.fieldtriptoolbox.org,^52^). First, data were down-sampled to 512 Hz, and filters were applied to decrease influence from high-frequency noise, slow wave drifts, and line noise (band-pass filer 0.1–70 Hz, and Notch filter at 50 Hz). Next, independent component analysis (ICA) was performed to correct for eye movement and blink artefacts. Artefacts caused by flat lines, jumps in the signal, muscles, clipping, or excessive noise were manually removed from the continuous data. Channels were removed from the data if artefacts affected more than 50% of the signal across the session. After data cleaning, the data were re-referenced to the average reference. This resulted in clean data segments of different lengths.

Next, we segmented the clean data segments into epochs of 1, 2, 3, 4, 5, and 6-s duration. We focussed on EEG connectivity in the alpha frequency band because this band displayed the highest test–retest reliability in the previous study, is characterised by a high signal-to-noise ratio, is less affected by muscle artefacts than other frequency bands, and is often the frequency band of interest in developmental studies^20,21,27,48,53,54^. Since alpha peaks typically occur at lower frequencies in younger participants, we selected our alpha band based on visual inspection of the power spectra calculated across the epochs from the first session for all 73 participants^21,53,55^. We observed a clear peak around 6–8 Hz (see Supplementary Information online), and selected these frequencies as the alpha band (consistent with ranges used in other studies in infants^21,51,56–58^).

### Selection of epoch lengths and numbers

In order to examine the biases towards epoch number, epoch length, and total data amounts, we selected different subsamples of the data for our calculation of EEG connectivity values. We took 3 approaches to selecting epochs and examining the reliability of subsamples: (1) *low numbers of longer epochs*: values across 20–60 epochs of 1–5 s duration each, with epochs randomly selected across each session^44,48^; (2) *high numbers of shorter epochs*: values across 30–150 epochs of 1 and 2 s duration each, with randomly selected epochs as in approach 1^21,27^; and (3) *constant total amount of data*: values across 120 1-s epochs, 60 2-s epochs, 40 3-s epochs, and 10 6-s epochs (where 10 6-s randomly selected epochs were segmented into 1-, 2-, and 3-s epochs to ensure that values for the different segmenting methods were calculated across the same data^21,45^). Only infants with artefact-free data across all 32 electrodes were included in these analyses, since connectivity metrics are influenced by the numbers of nodes and edges included in the networks^59^. Due to differences in amounts and lengths of artefact-free data for different infants, different subsamples were included for the different approaches: N_Low numbers of longer epochs_ = 19; N_High numbers of shorter epochs_ = 22; and N_Constant total amount of data_ = 41 (see Table 1, and Attrition rates in the Supplementary Information for a flow chart of the samples).

**Table 1:** Overview of subsets of data included in different analyses.

Analysis	Low numbers of longer epochs	High numbers of shorter epochs	Constant total amount of data
Segmenting combination^a^	20 × 1–2–3–4–5 s 30 × 1–2–3–4–5 s 40 × 1–2–3–4–5 s 50 × 1–2–3–4–5 s 60 × 1–2–3–4–5 s	30 × 1–2 s 60 × 1–2 s 90 × 1–2 s 120 × 1–2 s 150 × 1–2 s	120 × 1 s 60 × 2 s 40 × 3 s 20 × 6 s
N infants (males)	19 (7)	22 (7)	41 (16)
Age at session A^b^	302.3 (14.8) 279–342	303.8 (14.7) 279–342	301.7 (11.9) 279–342
Test–retest interval^b^	7.7 (1.8), 7–14	7.9 (2.4), 5–15	7.9 (3.2), 2–20

^a^Number × length (in s) of epochs included.

^b^Mean (sd), and range (in days).

### EEG connectivity measures of interest

The EEG connectivity measures of interest here were the PLI and dbWPLI. These measures were derived from the complex Fourier coefficients after applying a Fourier transform with a Hanning window to the epochs. We followed Vinck’s definition of the PLI and dbWPLI^44^:

For the PLI:

1 $$PLI=\left|E \left\{sgn\left(\mathfrak{I}\left\{X\right\}\right)\right\}\right|,$$

where I{X} is the imaginary component of the cross-spectrum, and E{.} is the expected value operator^44^.

For the dbWPLI:

2 $$WPLI= \frac{|E\left\{\mathfrak{I}\left\{X\right\}\right\}|}{E\{\left|\mathfrak{I}\left\{X\right\}\right|\}}= \frac{|E\left\{\left|\mathfrak{I}\left\{X\right\}\right|sgn\left(\mathfrak{I}\{X\right)\right\}|}{E\{\left|\mathfrak{I}\left\{X\right\}\right|\}},$$

where I{X} is the imaginary component of the cross-spectrum, and E{·} is the expected value operator^44^. We used in-house scripts to calculate Vinck’s PLI and dbWPLI values, which were identical to the ones used in^21,27^. PLI and dbWPLI-based connectivity matrices were averaged across the alpha frequency band (6–8 Hz). The matrices were subsequently used to calculate the network characteristics of interest: (a) whole brain connectivity, (b) the normalised weighted clustering coefficient, (c) the normalised weighted path length, and (d) the small-worldness index.

Whole brain connectivity was defined as the average (PLI or dbWPLI) connectivity across all possible electrode pairs.

Three further network characteristics were based on graph theory and calculated using Matlab functions and the Brain Connectivity Toolbox (BCT, available at https://sites.google.com/site/bctnet/)^60^ for the PLI values and absolute dbWPLI values^45^. Graph theory assumes that nodes (here, EEG sensors) are connected by edges with different values representing the strength of these connections (e.g., PLI or dbWPLI values)^60,61^. We computed weighted values rather than binary connectivity values, since thresholds for binary matrices are often arbitrarily chosen, and weak connections also provide information on the network^43^.

The normalised weighted clustering coefficient (C^w^_norm_) is a local metric reflecting functional segregation, and measures the average clustered connectivity around individual nodes^62,63^. We first calculated the average weighted clustering coefficient C^w^ across all 32 nodes (here, EEG channels) after rescaling the connection weights^62,63^:

3 $${C}^{w}= \frac{1}{n}\sum_{i\in N}\frac{2{t}_{ij}^{w}}{{k}_{i}({k}_{i}-1)},$$

We then computed C^w^_norm_ by dividing the observed clustering coefficient C^w^ from the weighted connectivity matrix by the average clustering coefficient C^w^_rand_ from 1,000 surrogate matrices^20^.

The normalised weighted path length (L^w^_norm_) is a global metric reflecting functional integration, and is measured as the average shortest path (sequence of edges) between two nodes^62^. We first calculated the observed weighted characteristic path length L^w^ after inversing the weights as the average shortest path lengths between nodes^62^:

4 $${L}^{w}= \frac{1}{n}\sum_{i\in N}\frac{{\sum }_{j\in N j\ne i}{d}_{ij}^{w}}{n-1},$$

The normalised path length or L^w^_norm_ was calculated as L^w^ divided by the average characteristic path length L^w^_rand_ across 1,000 surrogate connectivity matrices to obtain L^w^_norm_.

Finally, the small-worldness index (SWI) reflects the efficiency of the functional organisation of the network or graph, and is measured as the ratio between the normalised clustering coefficient and normalised characteristic path length^64^. We obtained values for the SWI by dividing the normalised weighted clustering coefficient by the normalised weighted path length^64^ as follows:

5 $$SWI= \frac{{C}_{norm}^{w}}{{L}_{norm}^{w}},$$

The results of these processing steps are 1 value for each of the 4 network characteristics (whole brain connectivity, normalised weighted clustering coefficient, normalised weighted path length, and small-worldness index), for both connectivity measures (PLI, and dbWPLI), for each session (test, and re-test), for each of the 3 approaches for individual infants.

### Statistical analyses

Test–retest reliability between the two sessions was calculated across participants using the intra-class correlation or ICC(3,1) (also called ICC(C-1)) with the following formula;

6 $$ICC\left(3,1\right)= \frac{{MS}_{R}-{MS}_{E}}{{MS}_{R}+\left(k-1\right){MS}_{E}},$$

where MS_R_ is between object variance (participant here), MS_E_ is the error variability or mean squared error, and *k* is the number of measurements per participant. The ICC (3,1) is a two-way fixed model ICC for single scores measuring consistency^65–67^, and has been used in previous test–retest reliability studies of EEG connectivity^38,45,48,49^. For ease of the reader, we use the term ICC to refer to ICC(3,1) here. We adapted the following convention to interpret the reliability values: poor—ICC < 0.40; fair—0.40 ≤ ICC ≤ 0.59; good—0.60 ≤ ICC ≤ 0.74; and excellent—ICC ≥ 0.75^35,38,45,49^. Negative ICC values were set to 0^42^. P values reflect whether the ICC value is significantly different from the null hypothesis. To further clarify, we are describing the pattern of ICC values, rather than statistically comparing ICC values with each other. Reliability of these measures not only depends on ICC values but also on the stability of the EEG measure and the aspect of connectivity being measured. Statistically comparing ICC values would falsely suggest that reliability differences depend on the number and lengths of epochs only. Therefore, we decided to describe the pattern of ICC values rather than statistically comparing the ICC values.

For conciseness, we only report ICC values for whole brain connectivity across low numbers of longer epochs, and high numbers of shorter epochs, and for graph metrics across a constant total amount of data which were based on different subsamples of the complete sample (see Table 1, Supplementary Tables S1–S3 online for original ICC values reported in the main manuscript, and Supplementary Tables S4–S9 online for reliability of graph metrics for low numbers of longer epochs, and for high numbers of shorter epochs).

## Results and discussion

### Reliability of whole brain connectivity across low numbers of longer epochs

**Figure 2:**
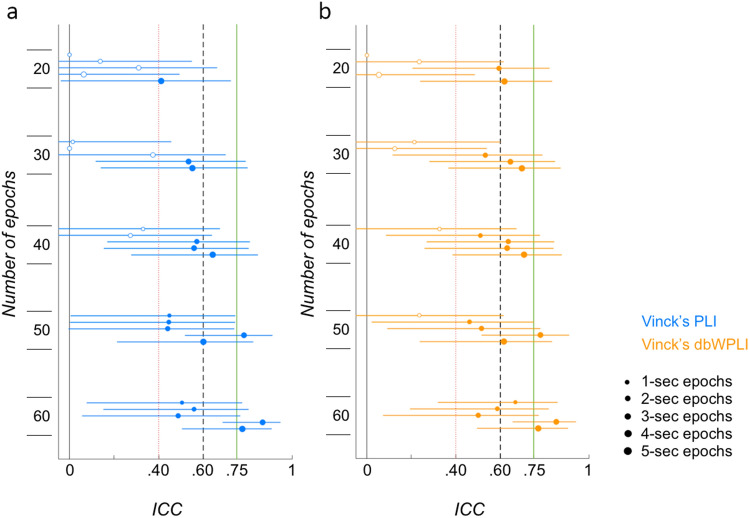
Intra-class correlations of whole brain connectivity for low numbers of longer epochs. ICC values increase with increasing epoch numbers and lengths for both Vinck’s PLI (**a**), and Vinck’s dbWPLI (**b**). Circles represent the ICC values (larger markers for increasing durations) that reached significance (p < 0.05, filled circles), or not (blank circles), with the lower and upper bound 95% confidence intervals (horizontal lines). Vertical lines represent the borders of the reliability ranges: poor—ICC < 0.40; fair—0.40 ≤ ICC ≤ 0.59; good—0.60 ≤ ICC ≤ 0.74; and excellent—ICC ≥ 0.75.

Figure 2 displays ICC values and their 95% confidence intervals across low numbers of longer epochs (N = 19). For the PLI-based whole brain connectivity, ICC values ranged from 0 to 0.87 (Fig. 2a). For the dbWPLI-based whole brain connectivity, ICC values ranged from 0 to 0.85 (Fig. 2b). ICC values generally increased with increasing epoch numbers and lengths. Reliabilities were within the poor range for 20 and 30 1- and 2-s epochs (0 ≤ ICC_PLI_ ≤ 0.14, 0 ≤ ICC_dbWPLI_ ≤ 0.24), and in the good and excellent ranges for 50 and 60 4- and 5-s epochs (0.60 ≤ ICC_PLI_ ≤ 0.87, 0.62 ≤ ICC_dbWPLI_ ≤ 0.85).

These findings suggest that (as might be expected) test–retest reliability in infants across a period of 1 week is higher when more data is included. M/EEG studies in adults found similar ICC values for connectivity in the good and excellent range. Whole brain connectivity based on PLI estimates from four 4-s epochs exhibited an ICC value of 0.61 for 8–10 Hz in an eyes-closed resting state paradigm assessed over a 2-year period^49^. Use of 12 4-s epochs for a whole brain PLI-based connectivity estimate showed excellent reliability with an ICC value of 0.79 for the same paradigm. The dbWPLI-based whole brain connectivity estimates were also highly reliable displaying an ICC value of 0.80^38^. In the infants, we observed similar values for 4-s epochs when calculated across at least 50 epochs for both the PLI- and dbWPLI-based measures. Thus, for infant studies more epochs are needed for reliable EEG connectivity estimates compared to adult studies. This moreover demonstrates that EEG methods typically applied in adults may not always be suitable for infant studies. Increased levels of noise in infant EEG data compared to adult EEG data are likely to play an important role in this difference.

Another possibility is that for infants a longer time of measurement is required to measure connectivity states that are stable across 1 week. Neuroimaging studies examining transient states of brain connectivity during rest and tasks suggest that the duration of brain states decreases and the number of transitions between brain states increases with development between childhood and adulthood (in EEG^68,69^, and fMRI studies^70–72^). If transient connectivity states exist for longer periods in infants compared to adults, then more time would be needed to pick up on these slower states compared to faster transient connectivity states in adults. In addition, developmental changes in connectivity strengths (both functional and structural) may also play a role here^70,73,74^. Stronger connectivity maps in adults may be better identifiable within a short time range compared to weaker, still developing connectivity maps in infants.

In comparison with our previous study^48^, current ICC values were lower than in the previous study when calculated across 20 5-s epochs (for the alpha1 band). The ICC_PLI_ was 0.41, [− 0.04, 0.72] (95% confidence interval) in the current study, and 0.84, [0.71, 0.92] in the previous study. The current ICC_dbWPLI_ was 0.62, [0.24, 0.83], while the previously found ICC_dbWPLI_ was 0.75, [0.54, 0.87]. One factor to take into account is the difference in the number of infants included in the sample. The requirement of a minimum of 60 epochs of 5-s duration significantly decreased the sample size from 60 to 19 infants in the present study. Smaller samples are less likely to detect a true large-sized effect than large samples^75^.

Another possible explanation for this discrepancy is that we used different pre-processing steps to calculate PLI- and dbWPLI-based connectivity measures. In our previous study, we derived the connectivity measures from instantaneous phase lags from a Hilbert transformation^46^, whereas we estimated phase lags from Fourier coefficients across epochs in the current study^44^. The Hilbert transform estimates instantaneous phases, but these estimates are more accurate for narrow band-pass filtered data compared to broad band-pass filtered data. Analyses across a broader frequency range would however include alpha peaks of more participants compared to analyses across a narrow frequency range. The method of Vinck et al.^44^ allows for the calculation of phase lag indices from the Fourier coefficients, and can be reliably calculated across a broader range of frequencies including the alpha peaks of different individuals as in the current study. The Fourier method thus may be more appropriate in research with developmental populations or a heterogeneous sample with high variability between individuals in alpha peaks^53,58,76,77^. Finally, use of the Fourier coefficients to estimate connectivity has previously led to replicable results in young infants^21,27^. These findings do suggest that when researchers want to estimate PLI-based connectivity for 20 5-s epochs, calculations from the narrow-band Hilbert transformed data are more reliable than calculations from the Fourier coefficients in homogeneous samples.

### Reliability of whole brain connectivity across high numbers of shorter epochs

**Figure 3:**
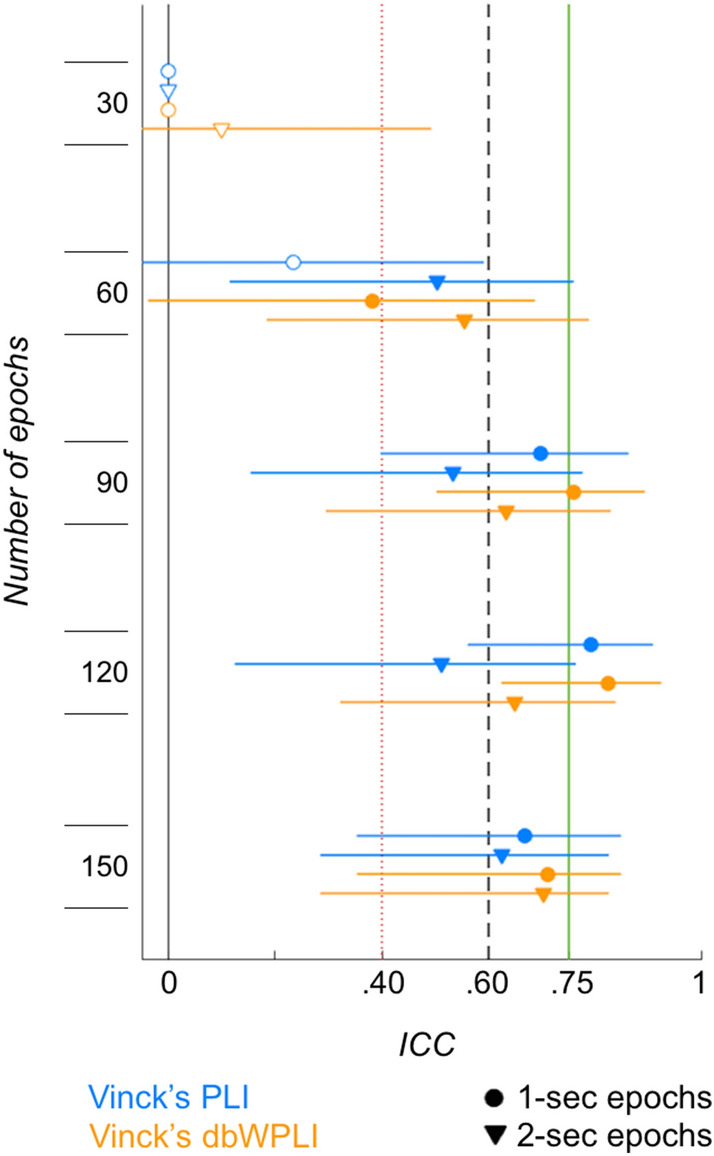
Intra-class correlations of whole brain connectivity for high numbers of short epochs. ICC values increase with increasing epoch numbers for both Vinck’s PLI (blue) and Vinck’s dbWPLI (orange). Furthermore, ICC values look higher for 1-s epochs (circles) than 2-s epochs (downward triangle). Markers represent ICC values that reached significance (p < 0.05, filled), or not (blank), with the lower and upper bound 95% confidence intervals (horizontal lines). Vertical lines represent the borders of the reliability ranges: poor—ICC < 0.40; fair—0.40 ≤ ICC ≤ 0.59; good—0.60 ≤ ICC ≤ 0.74; and excellent—ICC ≥ 0.75.

Results for the reliability analyses across high numbers of shorter epochs are depicted in Fig. 3 (N = 22). Again, ICC values increased with increasing numbers of epochs from poor reliability for 30 1- and 2-s epochs (0 ≤ ICCs ≤ 0.10) to good reliability for 150 1- and 2-s epochs (0.62 ≤ ICCs ≤ 0.71). With more than 90 epochs, ICC values seemed higher for 1- than 2-s epochs: for PLI-based connectivity across 1-s epochs, ICC_PLI_ = 0.70, 0.79, and 0.67, and for 2-s epochs, ICC_PLI_ = 0.53, 0.51, and 0.62, for 90, 120, and 150 epochs, resp.; and for dbWPLI-based connectivity across 1-s epochs, ICC_dbWPLI_ = 0.76, 0.82, and 0.71, and for 2-s epochs, ICC_dbWPLI_ = 0.63, 0.65, and 0.70, for 90, 120, and 150 epochs, respectively. Excellent reliability values were reached for dbWPLI-based connectivity across 90 and 120 1-s epochs, and for PLI-based connectivity across 120 1-s epochs. Across 120 1-s epochs, the ICC for dbWPLI-based connectivity was slightly higher than the ICC for PLI-based connectivity (ICC_dbWPLI_ = 0.82, versus ICC_PLI_ = 0.79).

These findings demonstrate that good and excellent reliable connectivity estimates can be achieved for 1- and 2-s epochs when calculated with the dbWPLI across at least 90 epochs, and with the PLI across at least 90 1-s and 150 2-s epochs. Consistent with the simulations from Vinck et al., the PLI and dbWPLI estimates show poor reliability when calculated across 30 1- or 2-s epochs^44^.

These results further suggest that reliability is higher for the 1-s compared to the 2-s epochs, and higher for the dbWPLI- than PLI-based whole brain connectivity. Two factors and their robustness to noise come to mind when explaining these findings. First, the assumption of stationarity of the signal for Fourier transform analysis may be violated for the different epoch lengths. The Fourier Transform assumes that the EEG signal can be decomposed into sines and cosines with a constant mean, variance, and covariance over time. This is more likely to hold true during shorter epochs of 1-s duration compared to epochs of 2-s duration, resulting in a more reliable estimate for shorter epochs^45,78^. Alternatively, estimates across longer epochs such as 5 s will even more likely show violations of non-stationarity. Indeed, we found lower ICC values for 20 5-s epochs than in our previous study where we derived our dbWPLI- and PLI-based estimates from Hilbert transformed data with instantaneous phase information instead of phase information from Fourier transformed data. Noise in the infant data will furthermore increase the non-stationarity of the signal, and thus amplify the effects of non-stationarity on the connectivity estimates across longer epochs.

Second, differences in reliability between the dbWPLI- and PLI-based estimates may arise from differences in robustness to noise. The dbWPLI weights the phase lag consistency such that phase differences near 0° or 180° angles contribute less to the final connectivity estimate than phase differences near 90° or 270° angles. Spurious connectivity values that may arise from noise with small phase differences are thus ignored^44^. The PLI in contrast does not apply these weights and is therefore less robust to noise artefacts. As expected for infant data with high noise levels^21,79^, the dbWPLI provides a more robust connectivity estimate than the PLI for these high numbers of shorter epochs when derived from Fourier coefficients.

### Reliability of network characteristics across a constant amount of data

**Figure 4:**
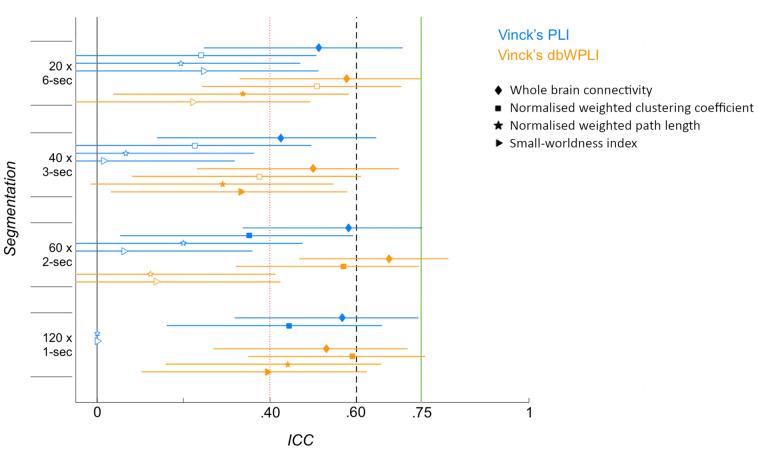
Intra-class correlations of connectivity metrics for different segmentation methods of a consistent total amount of data. For dbWPLI-based metrics (orange), ICC values are overall higher for 120 1-s epochs than for 20 6-s epochs for whole brain connectivity (diamond), normalised weighted clustering coefficient (square), normalised weighted path length (pentagram), and the small-worldness index (right-pointing triangle). For the PLI-based metrics (blue), ICC values for the different connectivity metrics were higher for 20 6-s epochs than 120 1-s epochs. Markers represent ICC values that reached significance (p < 0.05, filled), or not (blank), with the lower and upper bound 95% confidence intervals (horizontal lines). Vertical lines represent the borders of the reliability ranges: poor—ICC < 0.40; fair—0.40 ≤ ICC ≤ 0.59; good—0.60 ≤ ICC ≤ 0.74; and excellent—ICC ≥ 0.75.

Comparisons of the ICCs for different connectivity metrics across a constant amount of data are presented in Fig. 4 (N = 41). Across all segmentation and calculation methods, ICCs for whole brain connectivity were higher than ICCs for the other network characteristics (0.43 ≤ ICCs_Whole brain_ ≤ 0.86, and 0 ≤ ICCs_Graph metrics_ ≤ 0.59). ICCs for the normalised weighted clustering coefficient (0.23 ≤ ICCs ≤ 0.57) were higher than those for the normalised weighted path length (0 ≤ ICCs ≤ 0.44) and the small-worldness index (0 ≤ ICCs ≤ 0.40). For the dbWPLI-based metrics, the highest ICC for whole brain connectivity was found across 60 2-s epochs (ICC = 0.68), whereas ICCs for the other metrics were highest across 120 1-s epochs (ICC for C^w^_norm_ = 0.59, ICC for L^w^_norm_ = 0.44, and ICC for SWI = 0.40) compared to the other segmenting methods. For the PLI-based metrics, the highest ICC for whole brain connectivity was calculated across 60 2-s epochs (ICC = 0.58) compared to the other segmenting methods; for the normalised weighted clustering coefficient across 120 1-s epochs (ICC = 0.44); for the normalised weighted path length across 40 3-s epochs (ICC = 0.20); and for the small-worldness index across 20 6-s epochs (ICC = 0.25).

The current findings suggest that segmenting 2 min of EEG data into 1 or 2-s epochs provides more reliable dbWPLI-based connectivity metrics than segmenting into 3- or 6-s epochs. This was consistent with previous studies examining EEG connectivity in infants and adults^21,27,44,45^. Possibly, the debiasing and weighting methods are less robust to noise for low numbers compared to high numbers of epochs due to the normalisation or debiasing step that depends on the number of epochs^44^. Findings for the PLI-based connectivity metrics were however less consistent across segmentation methods, where the most reliable segmentation method varied with the connectivity metric of interest.

Furthermore, we found that whole brain connectivity was a more reliable metric than graph theory metrics (with the exception of the normalised clustering coefficient derived with the dbWPLI across 120 1-s epochs). Overall, the normalised weighted clustering coefficient showed more reliable estimates than the normalised weighted path length and the small worldness index. The observed pattern of reliabilities between connectivity metrics has been reproduced by several test–retest reliability studies in adults^35,36,38,42,49^. This pattern of increased reliability for first-order graph metrics compared to second-order metrics may arise from differences in variances in connectivity matrices where second-order graph theory metrics are more sensitive to variability in the connectivity matrices than first-order graph theory metrics^35^. Furthermore, it is possible that graph theory metrics cannot be reliably measured within these data segments, and more data (longer than 2 min in total) is needed to reliably measure graph metrics^42,80^.

Our previous study using the PLI across 20 5-s epochs showed a similar pattern between metrics: ICC = 0.84 for normalised clustering, ICC = 0.84 for the normalised path length, and ICC = 0.67 for the small-worldness-index^48^. As discussed in the previous section, the difference in ICC values between the previous and current study likely arises from the estimates of instantaneous phase differences with the Hilbert transform, and phase differences across the epochs with the Fourier transform.

We are currently unable to make comparisons with our previous findings for the graph metrics based on the dbWPLI. In our previous study, we found that inter-subject variability was higher, and that 95% confidence intervals were wider for dbWPLI-based than PLI-based whole brain connectivity. As a result, dbWPLI-based network characteristics were not included in further graph theory analyses. The current findings and previous simulations by Vinck et al.^44^ suggest that the number of 20 epochs may have been too low to calculate reliable dbWPLI-based network characteristics in infants.

## Conclusions

The current study demonstrates that EEG connectivity can be reliably estimated in young infants. Overall, reliability of EEG network characteristics increases with increasing total amounts of data. However, optimal epoch numbers and lengths for high test–retest reliability vary with the calculation method used to estimate EEG connectivity: smaller numbers of longer epochs for PLI-based measures, and higher numbers of shorter epochs for dbWPLI-based measures.

When choosing an EEG connectivity method in developmental research, several other factors need to be considered along with test–retest reliability. First, the quality of the EEG can have an impact on the reliability of EEG measures. For EEG data with lower noise levels and abundant lengths of artefact-free data, calculation of PLI-based whole brain connectivity from Hilbert transformed data across 20 5-s epochs would provide more reliable measures. For EEG data with higher noise levels and limited lengths of artefact-free data, dbWPLI-based whole brain connectivity from Fourier transformed data across more than 90 1-s or 60 2-s epochs would provide a reliable estimate of brain connectivity. The latter would be more appropriate in studies with vulnerable populations such as atypically developing young infants or individuals with neurodevelopmental disorders. Increased heterogeneity within such populations may also play a role.

Second, researchers should take into account the aspects of brain connectivity they aim to measure. Different EEG measures may be sensitive to different features of brain connectivity. Reliability estimates are influenced by both measurement error, and the stability of the process being measured over the selected timescale. Thus, one critical element to consider may be the timescale over which a particular measure of connectivity is stable. Within the present study, we examined reliability in infants tested twice with an average of a 1-week interval. Selection of this interval does lead to the possibility that there are true developmental changes in brain connectivity during the testing epoch. However, any decrease in interval may decrease the amount of artefact free data available, as infants may recognise repetition of the stimulus protocol and become less attentive (consistent with observations in the current study also). In a previous infant EEG study on event-related potentials, ICC values slightly *increased* when only including infants tested at intervals of 7 days or more, consistent with this possibility^34^. Of note, infant studies and longitudinal studies during early development often focus on age groups with a narrow range, commonly around 1–2 weeks. Measures that are stable over this interval are therefore necessary for data pooling. However, measures sensitive to more transient states of connectivity would appear unreliable in such an analysis, but this should not be taken as reflecting measurement noise. Some moment-to-moment fluctuations in connectivity may reflect shifts between cognitive states and may thus not be stable over time; researchers interested in individual differences in these states may need to derive higher level descriptions of their behaviour that do reflect persistent attributes, such as their intra-individual variability^71,72,81,82^. Researchers interested in a specific aspect of connectivity may wish to explore its reliability over several time intervals to dissociate measurement accuracy and developmental stability of different brain systems.

Finally, excellent test–retest reliability should be interpreted with caution. First, according to the paradox of reliability, excellently reliable and robust measures are unsuitable for correlational research: high test–retest reliability comes with low variability between individuals^83,84^. Excellently reliable measures that are stable over time reflect static constructs that are also likely stable in these individuals. The highly reliable construct however might not be the most relevant feature for brain-behaviour correlations (e.g. in fMRI research^85^). Thus, there is a dissociation between optimal test–retest reliability and their utility in predicting behaviour. This should especially be considered in the context of predictive biomarker research where the field is shifting from a categorical approach to a dimensional approach^83,86^. Second, high test–retest reliability values may be artificially increased by confounding factors that are stable themselves: such as head size, volume conduction, and measurement noise. It is possible that increased stable noise levels artificially increase the reliability of measures that are less robust to EEG noise (as in fMRI studies^87^). Thus, coupling the assessment of reliability with the assessment of robustness to time-invariant covariates (noise) is critical.

One limitation of this study is that only one age group was included in the current analyses. Reliability values and conclusions may differ for EEG data collected in toddlers or children compared to the data from 10-month-old infants in the current study. In addition, it is possible that conclusions vary between EEG data collected during the social and non-social dynamic videos^51^. Finally, we did not statistically compare the ICC values, but only tested whether the ICC values were different from the null hypothesis. Although methods exist to compare correlations, comparisons for ICC values are less straightforward as ICC values also depend on other factors such as stability of the EEG measure, measurement error, number and length of epochs. Here, we aimed to characterise the different comparison levels and explore the profile of EEG connectivity metrics.

Future research could consider reliability across different age groups and dynamic stimuli. Examining the reliability and the stability of brain connectivity at different age groups will further clarify whether early individual variability in brain connectivity persists into childhood and whether this is associated with later stable traits, for example restricted and repetitive behaviours in autism spectrum disorders^21,27^.

## Supplementary information

Supplementary file1 (PDF 548 kb)

## Data Availability

Data is available upon formal request from the YOUth Cohort Study, please see https://www.uu.nl/en/research/youth-cohort-study/data-access.

## References

[1] van den Heuvel MP, Sporns O (2019). A cross-disorder connectome landscape of brain dysconnectivity. Nat. Rev. Neurosci..

[2] Shen MD, Piven J (2017). Brain and behavior development in autism from birth through infancy. Dialogues Clin. Neurosci..

[3] Collin G, van den Heuvel MP (2013). The ontogeny of the human connectome: Development and dynamic changes of brain connectivity across the life span. Neuroscientist.

[4] Hoff GEA-J, Van den Heuvel MP, Benders MJNL, Kersbergen KJ, De Vries LS (2013). On development of functional brain connectivity in the young brain. Front. Hum. Neurosci..

[5] Menon V (2013). Developmental pathways to functional brain networks: Emerging principles. Trends Cogn. Sci..

[6] Vértes PE, Bullmore ET (2015). Annual research review: Growth connectomics—the organization and reorganization of brain networks during normal and abnormal development. J. Child Psychol. Psychiatry.

[7] Gao W (2019). A review on neuroimaging studies of genetic and environmental influences on early brain development. NeuroImage.

[8] Prince M (2007). No health without mental health. Lancet.

[9] Dasgupta J (2016). Translating neuroscience to the front lines: Point-of-care detection of neuropsychiatric disorders. Lancet Psychiatry.

[10] Keunen K, Counsell SJ, Benders MJNL (2017). The emergence of functional architecture during early brain development. Neuroimage.

[11] Turesky TK (2019). The relationship between biological and psychosocial risk factors and resting-state functional connectivity in 2-month-old Bangladeshi infants: A feasibility and pilot study. Dev. Sci..

[12] Omidvarnia A, Metsäranta M, Lano A, Vanhatalo S (2015). Structural damage in early preterm brain changes the electric resting state networks. Neuroimage.

[13] van den Heuvel MP (2014). The neonatal connectome during preterm brain development. Cereb. Cortex.

[14] Smyser CD, Wheelock MD, Limbrick DD, Neil JJ (2019). Neonatal brain injury and aberrant connectivity. NeuroImage.

[15] Smyser CD, Neil JJ (2015). Use of resting-state functional MRI to study brain development and injury in neonates. Semin. Perinatol..

[16] Gao W (2015). Functional network development during the first year: Relative sequence and socioeconomic correlations. Cereb. Cortex.

[17] Gao W (2011). Temporal and spatial evolution of brain network topology during the first two years of life. PLoS One.

[18] Gao W, Lin W, Grewen K, Gilmore JH (2017). Functional connectivity of the infant human brain. Neuroscience.

[19] O’Reilly C, Lewis JD, Elsabbagh M (2017). Is functional brain connectivity atypical in autism? A systematic review of EEG and MEG studies. PLoS One.

[20] Boersma M (2013). Disrupted functional brain networks in autistic toddlers. Brain Connect..

[21] Orekhova EV (2014). EEG hyper-connectivity in high-risk infants is associated with later autism. J. Neurodev. Disord..

[22] Righi G, Tierney AL, Tager-Flusberg HB, Nelson CA (2014). Functional connectivity in the first year of life in infants at risk for autism spectrum disorder: An EEG study. PLoS One.

[23] Murias M, Swanson JM, Srinivasan R (2007). Functional connectivity of frontal cortex in healthy and adhd children reflected in EEG coherence. Cereb. Cortex.

[24] Murias M, Webb SJ, Greenson J, Dawson G (2007). Resting state cortical connectivity reflected in EEG coherence in individuals with autism. Biol. Psychiatry.

[25] Ball G (2015). Thalamocortical connectivity predicts cognition in children born preterm. Cereb. Cortex.

[26] Alcauter S (2014). Development of thalamocortical connectivity during infancy and its cognitive correlations. J. Neurosci..

[27] Haartsen R (2019). Functional EEG connectivity in infants associates with later restricted and repetitive behaviours in autism; a replication study. Transl. Psychiatry.

[28] Fischi-Gómez E (2015). Structural brain connectivity in school-age preterm infants provides evidence for impaired networks relevant for higher order cognitive skills and social cognition. Cereb. Cortex.

[29] Harrop C (2014). Restricted and repetitive behaviors in autism spectrum disorders and typical development: Cross-sectional and longitudinal comparisons. J. Autism Dev. Disord..

[30] Shephard E (2020). Neural and behavioural indices of face processing in siblings of children with autism spectrum disorder (ASD): A longitudinal study from infancy to mid-childhood. Cortex.

[31] Fries P (2015). Rhythms for cognition: Communication through coherence. Neuron.

[32] Fries P (2005). A mechanism for cognitive dynamics: Neuronal communication through neuronal coherence. Trends Cogn. Sci..

[33] Lau-Zhu A, Lau MPH, McLoughlin G (2019). Mobile EEG in research on neurodevelopmental disorders: Opportunities and challenges. Dev. Cogn. Neurosci..

[34] Munsters NM, van Ravenswaaij H, van den Boomen C, Kemner C (2019). Test-retest reliability of infant event related potentials evoked by faces. Neuropsychologia.

[35] Deuker L (2009). Reproducibility of graph metrics of human brain functional networks. Neuroimage.

[36] Fraschini M (2016). The effect of epoch length on estimated EEG functional connectivity and brain network organization. J. Neural Eng..

[37] Miskovic V, Keil A (2015). Reliability of event-related EEG functional connectivity during visual entrainment: Magnitude squared coherence and phase synchrony estimates. Psychophysiology.

[38] Hardmeier M (2014). Reproducibility of functional connectivity and graph measures based on the phase lag index (PLI) and weighted phase lag index (wPLI) derived from high resolution EEG. PLoS One.

[39] Höller Y (2017). Reliability of EEG measures of interaction: A paradigm shift is needed to fight the reproducibility crisis. Front. Hum. Neurosci..

[40] Höller Y (2017). Reliability of EEG interactions differs between measures and is specific for neurological diseases. Front. Hum. Neurosci..

[41] Jin S-H, Seol J, Kim JS, Chung CK (2011). How reliable are the functional connectivity networks of MEG in resting states?. J. Neurophysiol..

[42] Moezzi B, Hordacre B, Berryman C, Ridding MC, Goldsworthy MR (2018). Test-retest reliability of functional brain network characteristics using resting-state EEG and graph theory. bioRxiv.

[43] van Diessen E (2015). Opportunities and methodological challenges in EEG and MEG resting state functional brain network research. Clin. Neurophysiol..

[44] Vinck M, Oostenveld R, Van Wingerden M, Battaglia F, Pennartz CMA (2011). An improved index of phase-synchronization for electrophysiological data in the presence of volume-conduction, noise and sample-size bias. Neuroimage.

[45] Kuntzelman K, Miskovic V (2017). Reliability of graph metrics derived from resting-state human EEG. Psychophysiology.

[46] Stam CJ, Nolte G, Daffertshofer A (2007). Phase lag index: Assessment of functional connectivity from multi channel EEG and MEG with diminished bias from common sources. Hum. Brain Mapp..

[47] Noreika V, Georgieva S, Wass S, Leong V (2020). 14 challenges and their solutions for conducting social neuroscience and longitudinal EEG research with infants. Infant Behav. Dev..

[48] van der Velde B, Haartsen R, Kemner C (2019). Test–retest reliability of EEG network characteristics in infants. Brain Behav..

[49] Hatz F (2016). Reliability of functional connectivity of electroencephalography applying microstate-segmented versus classical calculation of phase lag index. Brain Connect..

[50] Blasi A, Lloyd-Fox S, Johnson MH, Elwell C (2014). Test–retest reliability of functional near infrared spectroscopy in infants. Neurophotonics.

[51] Jones EJH, Venema K, Lowy R, Earl RK, Webb SJ (2015). Developmental changes in infant brain activity during naturalistic social experiences. Dev. Psychobiol.

[52] Oostenveld R, Fries P, Maris E, Schoffelen J-M (2011). FieldTrip: Open source software for advanced analysis of MEG, EEG, and invasive electrophysiological data. Comput. Intell. Neurosci..

[53] Shackman AJ, McMenamin BW, Maxwell JS, Greischar LL, Davidson RJ (2010). Identifying robust and sensitive frequency bands for interrogating neural oscillations. Neuroimage.

[54] Muthukumaraswamy SD (2013). High-frequency brain activity and muscle artifacts in MEG/EEG: A review and recommendations. Front. Hum. Neurosci..

[55] Saby JN, Marshall PJ (2012). The utility of EEG band power analysis in the study of infancy and early childhood. Dev. Neuropsychol..

[56] Stroganova TA, Orekhova EV, Posikera IN (1999). EEG alpha rhythm in infants. Clin. Neurophysiol..

[57] Orekhova EV, Stroganova TA, Posikera IN (2001). Alpha activity as an index of cortical inhibition during sustained internally controlled attention in infants. Clin. Neurophysiol..

[58] Marshall PJ, Bar-Haim Y, Fox NA (2002). Development of the EEG from 5 months to 4 years of age. Clin. Neurophysiol..

[59] van Wijk BCM, Stam CJ, Daffertshofer A (2010). Comparing brain networks of different size and connectivity density using graph theory. PLoS One.

[60] Rubinov M, Sporns O (2010). Complex network measures of brain connectivity: Uses and interpretations. Neuroimage.

[61] Bullmore E, Sporns O (2009). Complex brain networks: Graph theoretical analysis of structural and functional systems. Nat. Rev. Neurosci..

[62] Watts DJ, Strogatz SHH (1998). Collective dynamics of ‘small-world’ networks. Nature.

[63] Onnela JP, Saramäki J, Kertész J, Kaski K (2005). Intensity and coherence of motifs in weighted complex networks. Phys. Rev..

[64] Humphries MD, Gurney K (2008). Network ‘small-world-ness’: A quantitative method for determining canonical network equivalence. PLoS One.

[65] Shrout PE, Fleiss JL (1979). Intraclass correlations: Uses in assessing rater reliability. Psychol. Bull..

[66] Weir JP (2005). Quantifying test-retest reliability using the intraclass correlation coefficient and the SEM. J. Strength Cond. Res..

[67] Field AP, Everitt BS, Howell DC (2005). Intraclass correlation. Encyclopedia of Statistics in Behavioral Science, Vol 2.

[68] Koenig T (2002). Millisecond by millisecond, year by year: Normative EEG microstates and developmental stages. Neuroimage.

[69] Tomescu MI (2018). From swing to cane: Sex differences of EEG resting-state temporal patterns during maturation and aging. Dev. Cogn. Neurosci..

[70] Hutchison RM, Morton JB (2015). Tracking the brain’s functional coupling dynamics over development. J. Neurosci..

[71] Faghiri A, Stephen JM, Wang YP, Wilson TW, Calhoun VD (2018). Changing brain connectivity dynamics: From early childhood to adulthood. Hum. Brain Mapp..

[72] Rashid B (2018). Connectivity dynamics in typical development and its relationship to autistic traits and autism spectrum disorder. Hum. Brain Mapp..

[73] Grayson DS, Fair DA (2017). Development of large-scale functional networks from birth to adulthood: A guide to the neuroimaging literature. Neuroimage.

[74] Fair DA (2009). Functional brain networks develop from a ‘local to distributed’ organization. PLoS Comput. Biol..

[75] Button KS (2013). Power failure: Why small sample size undermines the reliability of neuroscience. Nat. Rev. Neurosci..

[76] Bazanova OM, Vernon D (2014). Interpreting EEG alpha activity. Neurosci. Biobehav. Rev..

[77] Dickinson A, DiStefano C, Senturk D, Jeste SS (2018). Peak alpha frequency is a neural marker of cognitive function across the autism spectrum. Eur. J. Neurosci..

[78] Cohen MX (2014). Analizing Neural Time Series Data: Theory and Practise.

[79] Goncharova II, McFarland DJ, Vaughan TM, Wolpaw JR (2003). EMG contamination of EEG: Spectral and topographical characteristics. Clin. Neurophysiol..

[80] Marquetand J (2019). Reliability of magnetoencephalography and high-density electroencephalography resting-state functional connectivity metrics. Brain Connect..

[81] Brookes MJ (2018). Altered temporal stability in dynamic neural networks underlies connectivity changes in neurodevelopment. Neuroimage.

[82] Falahpour M (2016). Underconnected, but not broken? Dynamic functional connectivity MRI shows underconnectivity in autism is linked to increased intra-individual variability across time. Brain Connect..

[83] Seghier ML, Price CJ (2018). Interpreting and utilising intersubject variability in brain function. Trends Cogn. Sci..

[84] Hedge C, Powell G, Sumner P (2017). The reliability paradox: Why robust cognitive tasks do not produce reliable individual differences. Behav. Res. Methods.

[85] Noble S (2017). Influences on the test–retest reliability of functional connectivity MRI and its relationship with behavioral utility. Cereb. Cortex.

[86] Insel T (2010). Research Domain Criteria (RDoC): Toward a new classification framework for research on mental disorders. Am. J. Psychiatry.

[87] Noble S, Scheinost D, Constable RT (2019). A decade of test-retest reliability of functional connectivity: A systematic review and meta-analysis. Neuroimage.

