# Supplementary material for: Using multiple short epochs optimises the stability of infant EEG connectivity parameters
Source: Sci Rep. 2020 Jul 29;10:12703. doi: 10.1038/s41598-020-68981-5 (PMC7391718; doi:10.1038/s41598-020-68981-5)
Supplement: Supplementary file 1 — Supplementary file1 (PDF 548 kb) [file 41598_2020_68981_MOESM1_ESM.pdf]

Supplementary Information:

Using multiple short epochs optimises the stability of infant EEG connectivity parameters

Rianne Haartsen, Bauke van der Velde, Emily J.H. Jones, Mark H. Johnson, & Chantal Kemner

Supplementary Methods

Layout of the EEG cap used

EEG was recorded with 32 electrodes with a Biosemi ActiveTwo system. The layout of the 32 electrodes and the placement of the CMS and DRL electrodes are displayed in Supplementary Figure S1.

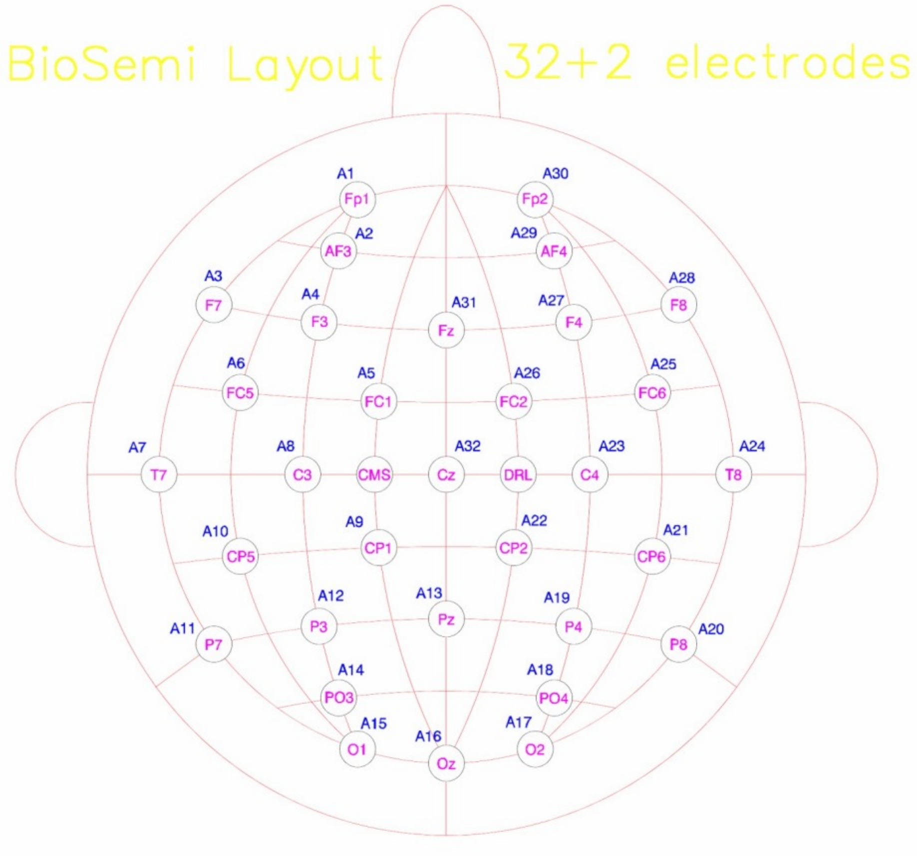

Supplementary Figure S1. EEG layout.

Selection of alpha frequency band

Peaks for the alpha band vary with age where alpha peaks are present at lower frequencies in younger participants (e.g. 6-9 Hz in 6- and 12-month-old infants) and at higher frequencies in older participants (e.g. 8-10 Hz in adults) [1–5]. We decided to take a data-driven approach and define our alpha frequency band by

examining the power spectrum rather than using a pre-defined alpha frequency band that are often based on either younger or older age groups. [2,6].

Spectral power was calculated from the Fast Fourier transform (Hanning window) by squaring the absolute FFT values, power across epochs, applying a natural log transform to the data, and finally, averaging the data across all electrodes. Mean and median power values across the group for 0 to 30 Hz for different epochs lengths are presented in Supplementary Figure S2. Two clear peaks are present across epoch duration: around 3 – 5 Hz, and around 6 – 8 Hz, which are the theta and alpha frequency peak, respectively. We thus decided to use 6-8 Hz as the alpha band for this 10-month-old infant dataset.

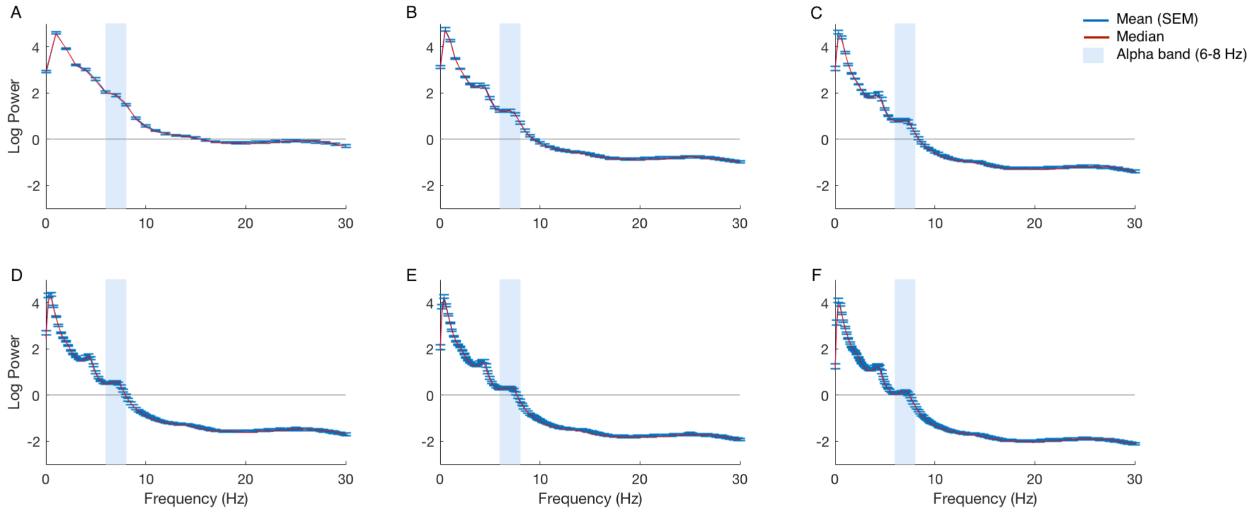

Supplementary Figure S2. Spectral power for 10-month-olds across different frequencies and epoch lengths
Log transformed power values (mean and standard error of mean in blue, and median in red) averaged across all available epochs for different lengths: 1, 2, 3, 4, 5, and 6 seconds in graphs a-f, resp. Frequencies range from 0 to 30 Hz. Light blue box marks the selected alpha frequency band from 6 to 8 Hz.

Attrition rates

Supplementary Figure S3 displays a flowchart of the samples tested and included in the previous test-retest reliability study and the subsamples included in each of the reliability analyses presented in the main manuscript.

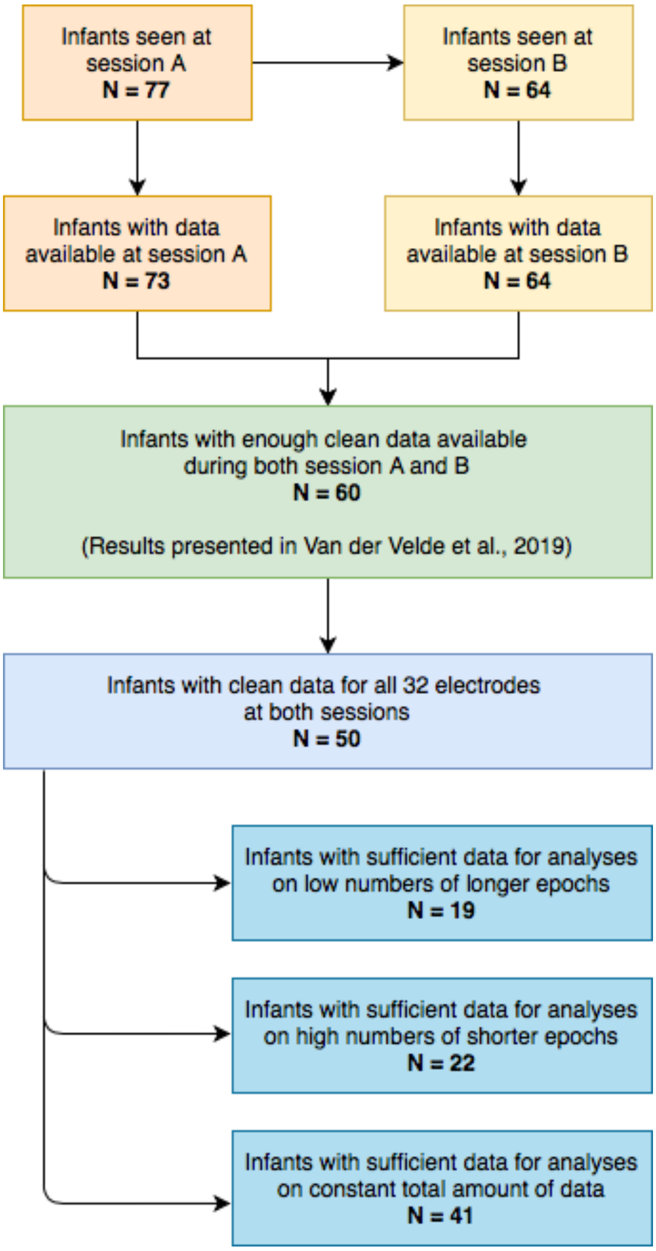

Supplementary Figure S3. Flowchart of included samples of the main analyses

In order to give a full characterisation of the data loss, we explored the number of artefact free 1-second epochs in session A and B from the 60 infants included in our previous study [7], and our current study. Supplementary figure S4 displays infant ages and the number of 1-second epochs available for the infants that were included in all analyses (for low numbers of longer epochs, high numbers of shorter epochs, and constant total amount of data; N = 19), 2 analyses (for high numbers of shorter epochs, and constant total amount of data; N = 3), 1 analysis (constant total amount of data; N = 19), or were excluded due to limited amounts of artefact free data (N = 9) or bad channels (N = 10). Note, even if datasets of infants would contain a high number of 1-second artefact free epochs, these infants may still be excluded because there were limited artefact free epochs of longer duration (e.g. 6-second duration).

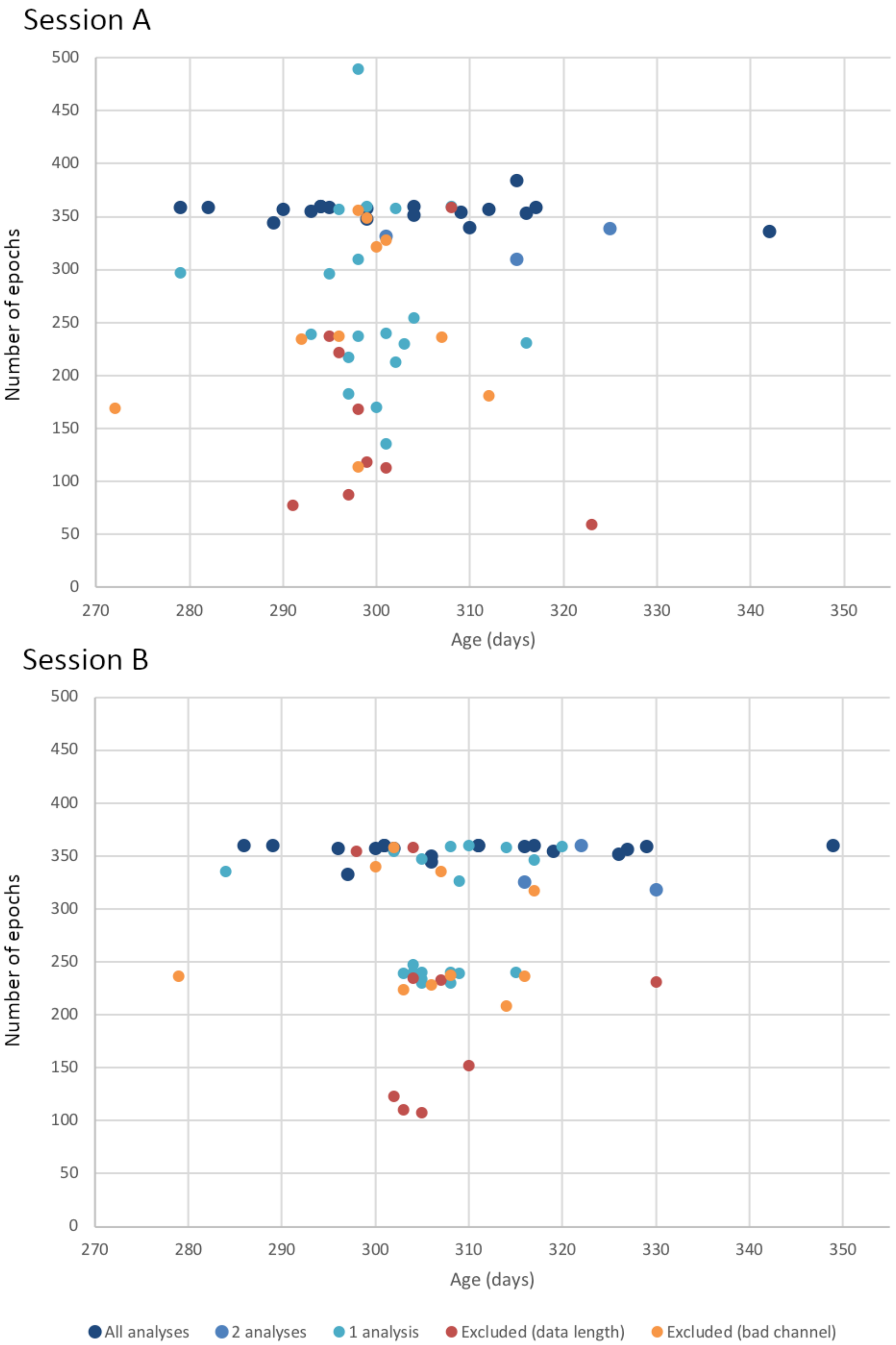

Supplementary figure S4. Ages at the time of different sessions and number of available 1-second artefact free epochs

Ages at the time of the session A (upper panel) and session B (lower panel) plotted against the number of 1-second epochs available for the infants that were included in all analyses (dark blue: for low numbers of longer epochs, high numbers of shorter epochs, and constant total amount of data), 2 analyses (mid blue; for high numbers of shorter epochs, and constant total amount of data), 1 analysis (light blue: constant total amount of data), or were excluded due to limited amounts of artefact free epochs (red) or bad channels (orange).

Our study was aimed at testing EEG connectivity with a test-retest interval of 1 week/ 7 days. We selected this interval because when protocols are repeated at too short an interval, data loss will be higher compared to protocols repeated at longer intervals. To further support this reasoning, we inspected the number of 1-second epochs in session B across the different testing intervals in Supplementary Figure S5. Any data loss due to data length occurred for datasets collected at intervals of 7 days or less. In contrast, datasets included in all 3 analyses or 2 analyses were collected at intervals of 5 days (for 1 infant) or 7 days or more (for 21 infants).

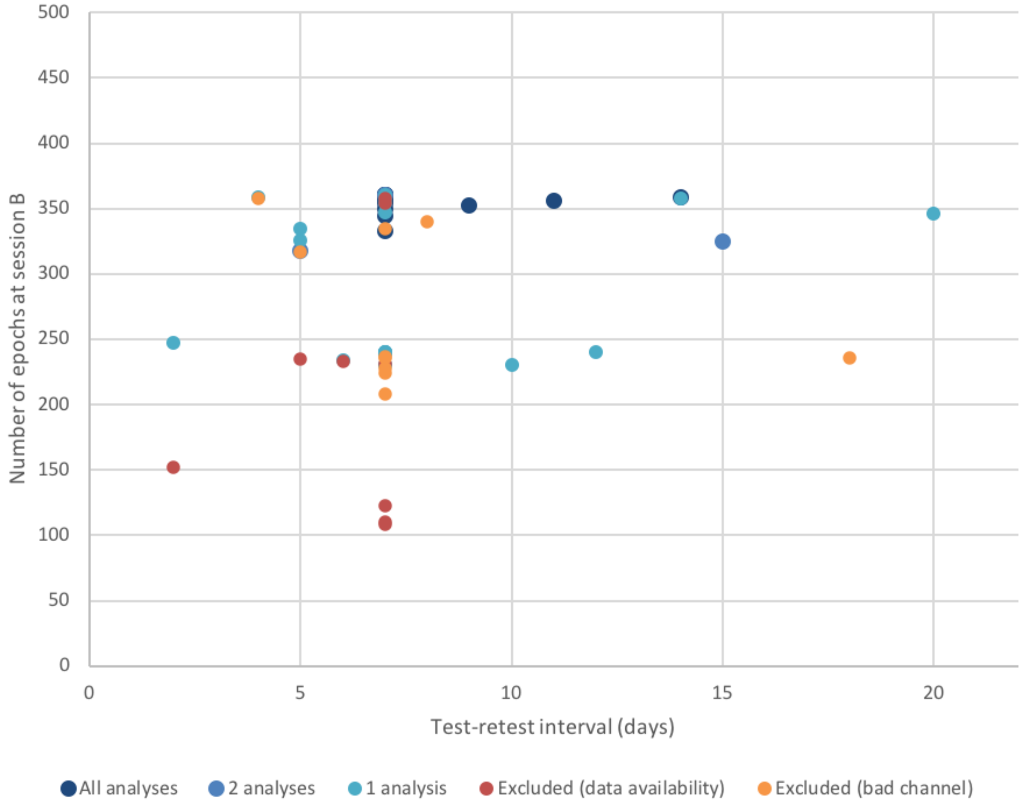

Supplementary figure S5. Test-retest intervals and number of available 1-second artefact free epochs in session B

Test-retest interval between session A and B (days) plotted against the number of 1-second epochs available in session B for the infants that were included in all analyses (dark blue: for low numbers of longer epochs, high numbers of shorter epochs, and constant total amount of data), 2 analyses (mid blue; for high numbers of shorter epochs, and constant total amount of data), 1 analysis (light blue: constant total amount of data), or were excluded due to limited amounts of artefact free epochs(red) or bad channels (orange).

Finally, we inspected the difference in epochs between session A and B with respect to the test-retest intervals. Supplementary Figure S6 shows the largest discrepancy differences in trial numbers occur for datasets collected at shorter intervals. This figure further reveals that several infants were only included in 1 analysis due to a large difference in epochs between sessions, mainly due to smaller numbers in session A compared to session B (reflected by negative values for the difference in numbers of epochs).

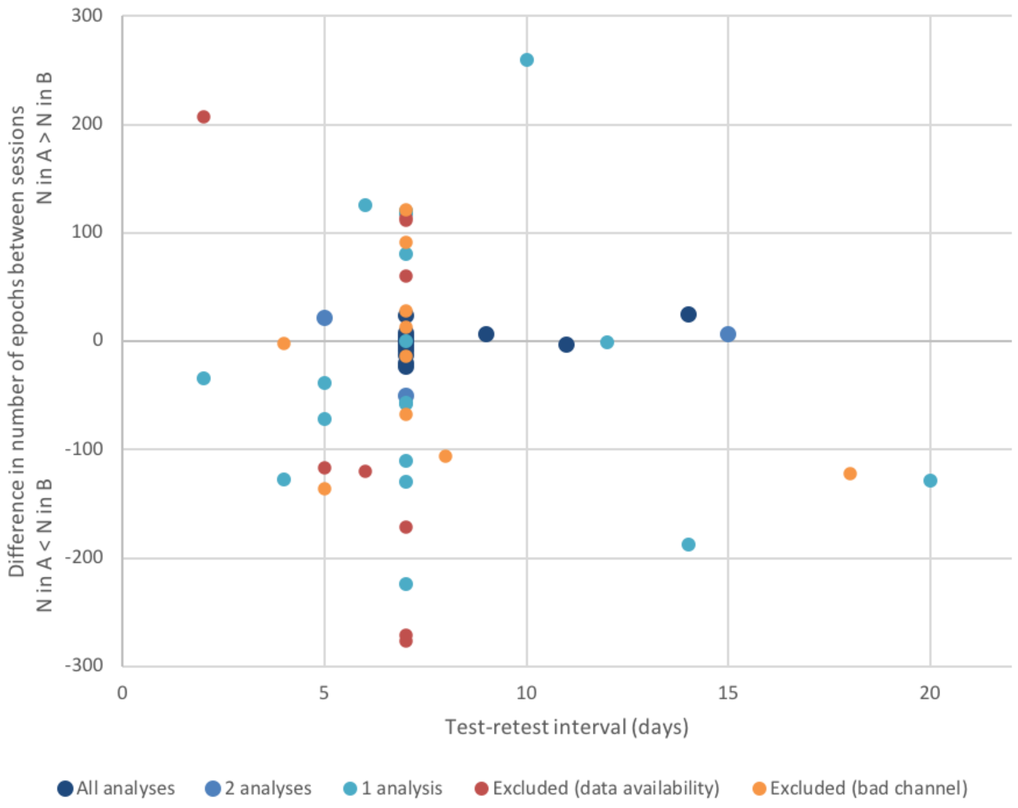

Supplementary figure S6. Test-retest intervals and differences in numbers of available 1-second artefact free epochs between session A and B
Test-retest interval between session A and B (days) plotted against the difference in numbers of 1-second epochs available at both sessions (N epochs in session A – N epochs in session B) for the infants that were included in all analyses (dark blue: for low numbers of longer epochs, high numbers of shorter epochs, and constant total amount of data), 2 analyses (mid blue; for high numbers of shorter epochs, and constant total amount of data), 1 analysis (light blue: constant total amount of data), or were excluded due to limited amounts of artefact free epochs (red) or bad channels (orange).

References to Supplementary Methods

1. Jones, E. J. H., Venema, K., Lowy, R., Earl, R. K. & Webb, S. J. Developmental changes in infant brain activity during naturalistic social experiences. *Dev. Psychobiol.* n/a-n/a (2015). doi:10.1002/dev.21336
2. Orekhova, E. V. *et al.* EEG hyper-connectivity in high-risk infants is associated with later autism. *J. Neurodev. Disord.* **6**, 1–11 (2014).
3. Stroganova, T. A., Orekhova, E. V. & Posikera, I. N. EEG alpha rhythm in infants. *Clin. Neurophysiol.* **110**, 997–1012 (1999).
4. Marshall, P. J., Bar-Haim, Y. & Fox, N. A. Development of the EEG from 5 months to 4 years of age. *Clin. Neurophysiol.* **113**, 1199–1208 (2002).
5. Saby, J. N. & Marshall, P. J. The Utility of EEG Band Power Analysis in the Study of Infancy and Early Childhood. *Dev. Neuropsychol.* **37**, 253–273 (2012).
6. Haartsen, R. *et al.* Functional EEG connectivity in infants associates with later restricted and repetitive behaviours in autism; a replication study. *Transl. Psychiatry* **9**, (2019).
7. van der Velde, B., Haartsen, R. & Kemner, C. Test-retest reliability of EEG network characteristics in infants. *Brain Behav.* **9**, e01269 (2019).

Supplementary Tables

ICC values for connectivity metrics reported in the main manuscript

In the main text, the ICC values and their 95% confidence intervals are presented in figures. We furthermore set the negative ICC values to 0 as these are not interpretable. In order to remain fully transparent, we decided to present all values in Supplementary Tables S1-3, including the negative values and additional thresholds of the p-values ($p < .05$, $p < .01$, and $p < .001$, instead of only $p < .05$ as in the main text).

Supplementary Table S1. Test-retest reliability of the PLI and dbWPLI-based whole brain connectivity for low numbers of longer epochs

	Epoch duration	Number of epochs				
		20	30	40	50	60
PLI	1 sec	**-.34** [-.68, .12]	**.02** [-.43, .46]	**.33** [-.13, .67]	**.45*** [.01, .74]	**.50*** [.08, .77]
	2 sec	**.14** [-.32, .55]	**-.06** [-.49, .39]	**.27** [-.20, .64]	**.45*** [.00, .74]	**.56**** [.15, .80]
	3 sec	**.31** [-.16, .66]	**.37** [-.08, .70]	**.57**** [.17, .81]	**.44*** [.00, .74]	**.49*** [.06, .77]
	4 sec	**.06** [-.39, .49]	**.53**** [.12, .79]	**.56**** [.15, .80]	**.78***** [.52, .91]	**.87***** [.69, .95]
	5 sec	**.41*** [-.04, .72]	**.55**** [.14, .80]	**.64**** [.28, .84]	**.60**** [.21, .82]	**.77***** [.50, .91]
dbWPLI	1 sec	**-.08** [-.51, .38]	**.21** [-.25, .60]	**.33** [-.14, .67]	**.24** [-.23, .62]	**.67***** [.32, .86]
	2 sec	**.24** [-.23, .62]	**.13** [-.34, .54]	**.51*** [.09, .78]	**.46*** [.02, .75]	**.59**** [.19, .82]
	3 sec	**.59**** [.21, .82]	**.53**** [.12, .79]	**.64**** [.27, .84]	**.51*** [.09, .78]	**.50*** [.07, .77]
	4 sec	**.05** [-.40, .49]	**.64**** [.28, .85]	**.63 **** [.26, .84]	**.78***** [.52, .91]	**.85***** [.66 - .94]
	5 sec	**.62**** [.24, .83]	**.70***** [.37, .87]	**.71***** [.39, .88]	**.62**** [.24, .83]	**.77***** [.50, .90]

Intraclass correlation in bold, and lower and upper bound of the 95% confidence

interval in square brackets below, * $p < .05$, ** $p < .01$, *** $p < 0.001$.
Coloured shading reflects the range for the ICC: red – poor, orange – fair, blue – good, and green – excellent ICC range.

Supplementary Table S2. Test-retest reliability of the PLI and dbWPLI-based whole brain connectivity for high numbers of shorter epochs

	Epoch duration	Number of epochs				
		30	60	90	120	150
PLI	1 sec	**-.21** [-.57, .22]	**.23** [-.20, .59]	**.70***** [.40, .86]	**.79***** [.56, .91]	**.67***** [.35, .85]
	2 sec	**0** [-.42, .41]	**.50**** [.11, .76]	**.53**** [.15, .78]	**.51**** [.12, .76]	**.62***** [.28, .82]
dbWPLI	1 sec	**-.03** [-.44, .39]	**.38*** [-.04, .69]	**.76***** [.50, .89]	**.82***** [.62, .92]	**.71***** [.42, .87]
	2 sec	**.10** [-.33, .49]	**.55**** [.18, .79]	**.63***** [.30, .83]	**.65***** [.32, .84]	**.70***** [.41, .86]

Intraclass correlation in bold, and lower and upper bound of the 95% confidence interval in square brackets below, * $p < .05$, ** $p < .01$, *** $p < 0.001$.
Coloured shading reflects the range for the ICC: red – poor, orange – fair, blue – good, and green – excellent ICC range.

Supplementary Table S3. Test-retest reliability of PLI- and dbWPLI based connectivity metrics across a constant total amount of data

Connectivity metric	Calculation method	Number and length of epochs			
		20 x 6 sec	40 x 3 sec	60 x 2 sec	120 x 1 sec
Whole brain connectivity	PLI	**.51***** [.25, .71]	**.43**** [.14, .65]	**.58***** [.34, .75]	**.57***** [.32, .74]
	dbWPLI	**.58***** [.33, .75]	**.50***** [.23, .70]	**.68***** [.47, .81]	**.53***** [.27, .72]
Normalised clustering coefficient	PLI	**.24** [-.07, .50]	**.23** [-.08, .50]	**.35*** [.05, .59]	**.44**** [.16, .66]
	dbWPLI	**.51***** [.24, .70]	**.38**** [.08, .61]	**.57***** [.32, .76]	**.59***** [.35, .76]
Normalised path length	PLI	**-.24** [-.51, .06]	**.20** [-.11, .48]	**.07** [-.24, .36]	**.19** [-.12, .47]
	dbWPLI	**.34*** [.04, .58]	**.29*** [-.01, .55]	**.12** [-.19, .41]	**.44**** [.16, .66]
Small-Worldness Index	PLI	**.25** [-.06, .51]	**.02** [-.29, .32]	**.06** [-.25, .36]	**-.22** [-.49, .09]
	dbWPLI	**.22** [-.09, .49]	**.33*** [.03, .58]	**.14** [-.17, .42]	**.40**** [.10, .62]

Intraclass correlation in bold, and lower and upper bound of the 95% confidence interval in square brackets below, * $p < .05$, ** $p < .01$, *** $p < 0.001$.
Coloured shading reflects the range for the ICC: red – poor, orange – fair, blue – good, and green – excellent ICC range.

ICC values for connectivity graph metrics for low numbers of longer epochs and high numbers of shorter epochs

The main text reports ICC values for whole brain connectivity across the segmenting methods for low numbers of longer epochs and high numbers of shorter epochs. The ICC values for the graph theory metrics for these segmentation methods are reported here for completeness in Supplementary Tables S4-9.

Supplementary Table S4. Test-retest reliability of the PLI and dbWPLI-based normalised clustering coefficient for low numbers of longer epochs

	Epoch duration	Number of epochs				
		20	30	40	50	60
PLI	1 sec	**.12** [-.35, .53]	**.27** [-.20, .64]	**.38*** [-.07, .71]	**-.12** [-.54, .34]	**.14** [-.32, .55]
	2 sec	**.30** [-.17, .65]	**-.16** [-.57, .30]	**-.10** [-.51, .36]	**.31** [-.16, .67]	**-.11** [-.53, .35]
	3 sec	**.10** [-.36, .52]	**.19** [-.28, .58]	**.15** [-.32, .55]	**-.14** [-.55, .32]	**.46*** [.02, .75]
	4 sec	**.13** [-.33, .54]	**.28** [-.19, .64]	**.39*** [-.07, .71]	**.54**** [.13, .79]	**.38** [-.08, .70]
	5 sec	**-.26** [-.63, .21]	**.22** [-.25, .60]	**.26** [-.20, .63]	**.43*** [-.02, .73]	**.38** [-.08, .70
dbWPLI	1 sec	**.09** [-.36, .51]	**.17** [-.29, .57]	**.15** [-.32, .56]	**-.08** [-.51, .37]	**.72***** [.40, .88]
	2 sec	**-.01** [-.57, .43]	**.24** [-.23, .62]	**.16** [-.31, .56]	**.43*** [-.01, .74]	**.46*** [.02, .75]
	3 sec	**.58**** [.18, .81]	**.43*** [-.02, .73]	**.26** [-.20, .63]	**.38*** [-.07, .71]	**.25** [-.21, .63]
	4 sec	**.22** [-.24, .61]	**.36** [-.10, .70]	**.31** [-.16, .66]	**.65**** [.29, .85]	**.67***** [.32, .86]
	5 sec	**.57**** [.17, .81]	**.69***** [.35, .86]	**.41*** [-.05, .72]	**.52**** [.10, .78]	**.36** [-.10, .70]

Intraclass correlation in bold, and lower and upper bound of the 95% confidence

interval in square brackets below, * *p* < .05, ** *p* < .01, *** *p* < 0.001.
Coloured shading reflects the range for the ICC: red – poor, orange – fair, blue – good, and green – excellent ICC range.

Supplementary Table S5. Test-retest reliability of the PLI and dbWPLI-based normalised path length for low numbers of longer epochs

	Epoch duration	Number of epochs				
		20	30	40	50	60
PLI	1 sec	**.02** [-.43, .46]	**-.04** [-.48, .41]	**-.11** [-.53, .35]	**-.34** [-.68, .12]	**-.05** [-.48, .40]
	2 sec	**-.09** [-.51, .37]	**-.01** [-.45, .44]	**-.31** [-.66, .16]	**.20** [-.27, .59]	**.17** [-.30, .57]
	3 sec	**-.27** [-.64, .20]	**-.14** [-.55, .32]	**.18** [-.29, .58]	**-.24** [-.62, .23]	**-.09** [-.52, .37]
	4 sec	**.40*** [-.06, .72]	**.40*** [-.05, .72]	**.17** [-.30, .57]	**.47*** [.04, .76]	**.30** [-.16, .66]
	5 sec	**-.04** [-.48, .41]	**-.23** [-.61, .24]	**-.15** [-.56, .31]	**.38** [-.08, .70]	**.18** [-.28, .58]
dbWPLI	1 sec	**-.09** [-.51, .37]	**-.08** [-.51, .38]	**-.02** [-.46, .43]	**.32** [-.14, .67]	**.08** [-.38, .51]
	2 sec	**.41*** [-.05, .72]	**-.19** [-.58, .28]	**-.08** [-.50, .38]	**.43*** [-.02, .73]	**.15** [-.31, .56]
	3 sec	**-.34** [-.68, .12]	**-.19** [-.58, .28]	**.64**** [.27, .84]	**-.14** [-.55, .32]	**-.20** [-.59, .27]
	4 sec	**.10** [-.36, .52]	**.13** [-.34, .54]	**.41*** [-.04, .72]	**.19** [-.28, .59]	**.55**** [.14, .80]
	5 sec	**.42*** [-.02, .73]	**.33** [-.13, .68]	**-.21** [-.60, .26]	**.35** [-.11, .69]	**.19** [-.28, .58]

Intraclass correlation in bold, and lower and upper bound of the 95% confidence interval in square brackets below, * *p* < .05, ** *p* < .01, *** *p* < 0.001.
Coloured shading reflects the range for the ICC: red – poor, orange – fair, blue – good, and green – excellent ICC range.

Supplementary Table S6. Test-retest reliability of the PLI and dbWPLI-based Small-Worldness Index for low numbers of longer epochs

	Epoch duration	Number of epochs				
		20	30	40	50	60
PLI	1 sec	**-.00** [-.44, .44]	**-.02** [-.46, .43]	**-.10** [-.52, .36]	**-.36** [-.70, .10]	**-.11** [-.53, .35]
	2 sec	**-.20** [-.60, .27]	**.04** [-.41, .48]	**-.29** [-.65, .17]	**.27** [-.20, .64]	**.10** [-.36, .52]
	3 sec	**-.24** [-.62, .23]	**-.08** [-.51, .36]	**.21** [-.26, .60]	**-.31** [-.66, .15]	**-.20** [-.59, .27]
	4 sec	**.35** [-.11, .69]	**.36** [-.10, .69]	**.15** [-.31, .56]	**.57**** [.17, .81]	**.42*** [-.02, .73]
	5 sec	**.06** [-.40, .49]	**-.19** [-.58, .28]	**-.07** [-.50, .38]	**.45*** [.01, .75]	**.33** [-.14, .67]
dbWPLI	1 sec	**-.07** [-.50, .39]	**-.11** [-.53, .35]	**-.19** [-.58, .28]	**.26** [-.21, .63]	**.07** [-.39, .50]
	2 sec	**.25** [-.22, .62]	**-.04** [-.47, .41]	**.01** [-.44, .45]	**.54**** [.12, .79]	**.25** [-.22, .62]
	3 sec	**-.39** [-.71, .06]	**-.09** [-.51, .37]	**.64**** [.27, .84]	**-.03** [-.46, .42]	**-.32** [-.67, .14]
	4 sec	**.19** [-.27, .59]	**.22** [-.24, .61]	**.39*** [-.07, .71]	**.30** [-.16, .66]	**.60**** [.21, .82]
	5 sec	**.34** [-.12, .68]	**.32** [-.14, .67]	**-.10** [-.52, .36]	**.40*** [-.05, .72]	**.35** [-.11, .69]

Intraclass correlation in bold, and lower and upper bound of the 95% confidence interval in square brackets below, * $p < .05$, ** $p < .01$, *** $p < 0.001$.
Coloured shading reflects the range for the ICC: red – poor, orange – fair, blue – good, and green – excellent ICC range.

Supplementary Table S7. Test-retest reliability of the PLI and dbWPLI-based normalised clustering coefficient for high numbers of shorter epochs

	Epoch duration	Number of epochs				
		30	60	90	120	150
PLI	1 sec	**.20**<br>[-.23, .57]	**.08**<br>[-.34, .48]	**.20**<br>[-.23, .56]	**.27**<br>[-.16, .62]	**.32**<br>[-.11, .65]
	2 sec	**.21**<br>[-.22, .58]	**.14**<br>[-.29, .52]	**-.05**<br>[-.46, .37]	**.29**<br>[-.14, .63]	**-.03**<br>[-.44, .39]
dbWPLI	1 sec	**.22**<br>[-.22, .58]	**.30**<br>[-.13, .63]	**.59****<br>[.24, .80]	**.53****<br>[.15, .78]	**.48***<br>[.08, .74]
	2 sec	**.37***<br>[-.05, .68]	**.32**<br>[-.10, .65]	**.42***<br>[.01, .71]	**.49****<br>[.10, .75]	**.27**<br>[-.16, .61]

Intraclass correlation in bold, and lower and upper bound of the 95% confidence interval in square brackets below, * $p < .05$, ** $p < .01$, *** $p < 0.001$.
Coloured shading reflects the range for the ICC: red – poor, orange – fair, blue – good, and green – excellent ICC range.

Supplementary Table S8. Test-retest reliability of the PLI and dbWPLI-based normalised path length for high numbers of shorter epochs

	Epoch duration	Number of epochs				
		30	60	90	120	150
PLI	1 sec	**.01**<br>[-.40, .42]	**.04**<br>[-.38, .44]	**.01**<br>[-.41, .42]	**.26**<br>[-.17, .61]	**-.04**<br>[-.44, .38]
	2 sec	**-.21**<br>[-.57, .22]	**.30**<br>[-.13, .63]	**.10**<br>[-.33, .49]	**.37***<br>[-.04, .68]	**.20**<br>[-.23, .57]
dbWPLI	1 sec	**-.08**<br>[-.48, .35]	**.38***<br>[-.04, .68]	**-.18**<br>[-.55, .25]	**.30**<br>[-.13, .63]	**.34**<br>[-.09, .66]
	2 sec	**-.13**<br>[-.52, .30]	**.09**<br>[-.34, .48]	**.46***<br>[.06, .73]	**.41***<br>[-.01, .70]	**.35***<br>[-.07, .67]

Intraclass correlation in bold, and lower and upper bound of the 95% confidence interval in square brackets below, * $p < .05$, ** $p < .01$, *** $p < 0.001$.
Coloured shading reflects the range for the ICC: red – poor, orange – fair, blue – good, and green – excellent ICC range.

Supplementary Table S9. Test-retest reliability of the PLI and dbWPLI-based Small-Worldness Index for high numbers of shorter epochs

	Epoch duration	Number of epochs				
		30	60	90	120	150
PLI	1 sec	**-.02**<br>[-.43, .39]	**.08**<br>[-.34, .48]	**-.16**<br>[-.53, .27]	**.33**<br>[-.09, .66]	**.07**<br>[-.35, .47]
	2 sec	**-.20**<br>[-.57, .23]	**.31**<br>[-.12, .64]	**.10**<br>[-.32, .49]	**.34**<br>[-.08, .66]	**.20**<br>[-.23, .57]
dbWPLI	1 sec	**-.06**<br>[-.46, .36]	**.46***<br>[.05, .73]	**-.07**<br>[-.47, .35]	**.31**<br>[-.10, .65]	**.41***<br>[0, .71]
	2 sec	**-.05**<br>[-.45, .37]	**.13**<br>[-.30, .51]	**.40***<br>[-.02, .70]	**.37***<br>[-.05, .68]	**.36***<br>[-.07, .67]

Intraclass correlation in bold, and lower and upper bound of the 95% confidence interval in square brackets below, * $p < .05$, ** $p < .01$, *** $p < 0.001$.
Coloured shading reflects the range for the ICC: red – poor, orange – fair, blue – good, and green – excellent ICC range.
